# The condition of the oral cavity at the time of diagnosis of inflammatory bowel disease in pediatric patients

**DOI:** 10.1038/s41598-021-01370-8

**Published:** 2021-11-09

**Authors:** Małgorzata Klichowska-Palonka, Aneta Komsta, Elżbieta Pac-Kożuchowska

**Affiliations:** 1 Independent Department of Integrated Dentistry, Medical University of Lublin, Lublin, Poland; 2 Chair and Department of Conservative Dentistry with Endodontics, Medical University of Lublin, Lublin, Poland; 3 Department of Paediatrics and Gastroenterology, Medical University of Lublin, Lublin, Poland

**Keywords:** Diseases, Gastroenterology, Signs and symptoms

## Abstract

Changes in the oral mucosa can appear in the course of inflammatory bowel disease in both children and adults. They often precede the appearance of gastrointestinal symptoms. The aim of the study was to determine the nature of changes in the oral cavity at the time of diagnosis of inflammatory bowel disease in children compared to children without systemic diseases. 49 children diagnosed with inflammatory bowel disease and 60 children without systemic diseases were examined. The prevalence of the aphthae stomatitis and angular cheilitis was 24.5% in the examined group and 10% in the control group (*p* = 0.0772). Changes in the oral mucosa occurred more frequently in children with Crohn's disease 35.3% than with ulcerative colitis 18.7%. In children with Crohn's disease, the most frequently observed lesion was aphthous stomatitis 23.5%, and in ulcerative colitis, angular cheilitis 12.5%. Changes in the oral mucosa are a therapeutic problem requiring in general diseases patients both local and systemic treatment and interdisciplinary cooperation between dentists, paediatricians and gastroenterologists. The finding of repeated changes in the oral mucosa during a dental examination should be the reason for referring the patient to a paediatrician for the foreclosure or make a diagnosis of inflammatory bowel diseases.

## Introduction

The oral cavity may be the first site of mucosal changes that may represent local mucosal disease and systemic conditions or be part of a broader systemic involvement. Known diseases of body systems that manifest in mucosal/gingival changes are for example: blood disorders, connective tissue diseases, endocrine diseases, dermatologic diseases and gastrointestinal diseases^1^. Gastrointestinal diseases such as Crohn's disease (CD) and ulcerative colitis (UC) belong to the group of inflamatory bowel disease. Inflammatory bowel disease (IBD) is disease of unknown multifactorial etiology. In their development, factors such as: immunoregulation disorders in the intestinal mucosa with increased activity of T-helper lymphocytes, infections, genetic conditions and environmental factors are all taken into account^2^. In the course of chronic inflammatory bowel disease, in addition to gastrointestinal symptoms, there may occur parenteral symptoms affecting the skin, eyes, joints and the mouth. Oral lesions include recurrent mouth sores, erosions, crater-like ulcers, cheilitis, enlarged warts, facial edema, atrophic mucositis, and oral mycoses. Gastrointestinal symptoms in IBD may be preceded by the appearance of lesions in the oral cavity^3–5^. Oral manifestations were divided on specific and non-specific. Specific oral manifestations in patients with CD are as follows: indurate mucosal tags, cobblestoning and mucogingivitis, deep linear ulcerations and lip swelling with vertical fissures. The most common non-specific manifestations, such as aphthous stomatitis and angular cheilitis, occur in both IBD diseases^3^. *Pyostomatitis vegetans* is considered as a specific marker of disease activity of the UC^1,6^. Changes in the oral mucosa are more common in CD than in UC.

There exist reports that describe the relationship between the severity of caries or gingivitis and the activity status of the disease process in patients with inflammatory bowel disease, which may be related to the composition of saliva, colonization with specific bacteria strains or the diet used^2,7,8^. There are examples that connecting oral lesions to early presentation of systemic conditions potentially reduces a patient's systemic disease burden and improves their quality of life^9^.

The aim of the study was to determine the nature of the changes occurring in the oral cavity at the time of diagnosis of inflammatory bowel disease in children compared to children without systemic diseases.

## Materials and methods

Each children presenting with suspected IBD to the Department of Paediatrics and Gastrology were examined by dentist. Data including age, sex, oral symptoms, family history, and medications taken, were collected. Clinical dental examination were performed. After received positive results of diagnostic tests for CD or UC children were included in the study group. The study group included 49 patients with inflammatory bowel disease, 25 girls, 24 boys, aged 6 to 17 years old, 16 patients diagnosed with Crohn's disease and 33 patients with ulcerative colitis. CD activity was assessed using the PCDAI scale (pediatric Crohn's diseases activity index) and UC disease using the PUCAI scale (pediatric ulcerative colitis activity index). In the studied group of children, most of the changes were mild and moderate. The distribution of lesions was determined using the Paris scale. The large intestine was partially or fully affected in most patients with CD, and the upper gastrointestinal tract was affected in individual cases. In the majority of children diagnosed with UC, the lesions affected the entire colon, and in individual cases the lesions were limited to the rectum. All oral cavity examinations were performed before the beginning of treatment of IBD. Generally healthy children of similar age and gender were recruited into the control group. The interview and examination were conducted in a dentist's office. The control group consisted of 60 children (29 girls and 31 boys) without systemic diseases. The age and gender of children in the study and control groups did not differ statistically. According to the guidelines of the bioethics committee, the parents of all patients gave their written, informed consent for their children to participate in the research. Patients aged 16 and over also signed an additional informed consent for the study. In both groups of patients, the condition of the oral mucosa were examined and changes in the following clinical features were taken into account: colour, texture and structure. Every observed changes were recorded in to research card. The statistical evaluation included all the observed changes as well as changes considered to be associated with IBD. The condition of the dentition was assessed by calculating the frequency and intensity of caries, the average number of DMFT and its components. Periodontal examination was performed using the community periodontal index of treatment needs (CPITN for six index teeth) as an epidemiological screening procedure for monitoring persons under 20 years of age. The study of patients with IBD was carried out in the hospital ward by two experienced dentists using the same assessment criteria. The parents were present during the study. All observations were noted manually on a specially developed research card. Data was anonymized during collection (no sensitive personal information such as surname or date of birth was collected). The participants of the control group were examined by the same doctors according to the same criteria in the dentist's office.

### Statistical analysis

The following tests were used to perform the statistical analysis: Kolmogorov–Smirnov test, age—control vs tested Student's t-test, other comparisons (D, F, DMFT): Mann–Whitney test, assessment of differences for the qualitative traits chi-2 and chi-2 Yates tests (Tables 3, 4). Statistical analysis was performed using the Statistica 13.3 program, assuming statistically significant differences for *p* < 0.05. The study was approved by the Bioethics Committee of Medical University of Lublin no. KE-0254/155/2014.

### Ethical approval

All procedures performed in studies involving human participants were in accordance with the ethical standards of the institutional and national research committee and with the 1964 Helsinki declaration and its later amendments or comparable ethical standards.

### Informed consent

Informed consent was obtained from all individual participants included in the study.

## Results

In our study, the group of children and adolescents included in the study were patients diagnosed with ulcerative colitis or Crohn's disease during hospitalization. In the study group, 32 patients (65.4%) were diagnosed with UC, while CD was diagnosed in 17 patients (34.6%). The caries frequency in the patients with IBD was 84%, and the mean DMFT number was 3.82 ± 3.07, in the control group it was 70% and mean DMFT 4.0 ± 4.08. The value of the DMFT index and its components did not differ significantly between the study and the control group. (Table 1). Patients from the study group and the control group haven't missing teeth due to caries (M = 0).

**Table 1:** Characteristics sex, changes in the oral mucosa and tooth caries in the study and control groups.

	Study group		Control group		*p*
	n	%	n	%	
Female	25	51.0	29	48.3	n.s
Male	24	49.0	31	51.7	

Type of changes	Study group		Control group		*p*
	n	%	n	%	
No changes	36	73.5	50	83.3	n.s
Lips herpes	1	2.1	4	6.7	
Aphthae stomatitis	6	12.2	4	6.7	
Angular cheilitis	6	12.2	2	3.3	

Caries Index	Study group		Control group		*p*
	M	SD	M	SD	
Decayed Teeth (D)	1.51	2.15	1.35	2.20	n.s
Filled Teeth (F)	2.31	2.11	2.65	2.80	n.s
DMFT Index	3.82	3.07	4.00	4.08	n.s

Healthy periodontium was found in 57.1% of the patients in the study group and 66.7% of children in the control group. Bleeding during probing the gingival fissure was observed in 14.3% of patients in the test group and in 6,6% of the control group, tartar in 28.6% in the study group and 26.7% of the control group. Periodontal status did not differ significantly between the examined groups (Table 2).

**Table 2:** Periodontal status in the study and control groups.

Periodontal status	Study group		Control group		*p*
	n	%	n	%	
Healthy periodontium	28	57.1	40	66.7	n.s
Gum bleeding	7	14.3	4	6.6	
Tartar	14	28.6	16	26.7	

All lips and mucosa changes were recorded. The following changes were observed: herpes on the lips, aphthae, angular cheilitis. 26.5% of patients in the study group were changes within the oral mucosa. The following changes were observed: aphthae stomatitis, angular cheilitis, lips herpes and their percentage was correspondingly 12.2%; 12.2%; 2.1%. In the control group 16.7% of patients had mucosa changes: herpes in 6.7%, aphthae in 6.7% and angular cheilitis 3.33% of patients. There were no statistically significant differences in the occurrence of mucosal lesions in the study group compared to the control group (Table 1). Taking into account the changes in the oral mucosa accompanying IBD, only two nonspecific manifestations: angular cheilitis and aphthous ulcerations were found. In the study group nonspecific manifestations of IBD in the oral mucosa occurred in 24.4% of patients and only in 10% of the healthy children, however, there are no significant differences between the groups (*p* = 0.07) (Table 3).

**Table 3:** Nonspecific manifestations of IBD in the oral mucosa in the study and control groups.

Manifestations of IBD	Study group		Control group		*p*
	n	%	n	%	
No changes	37	75.5	54	90.0	0.0772
Aphthae stomatitis and angular cheilitis	12	24.5	6	10.0	

**Figure 1:**
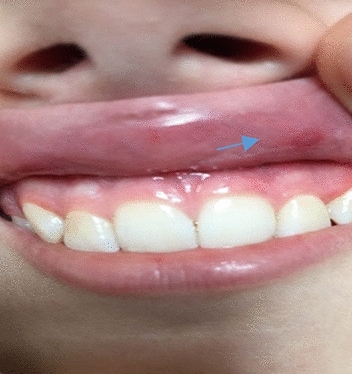
Aphthae in a child diagnosed with Crohn's disease.

**Figure 2:**
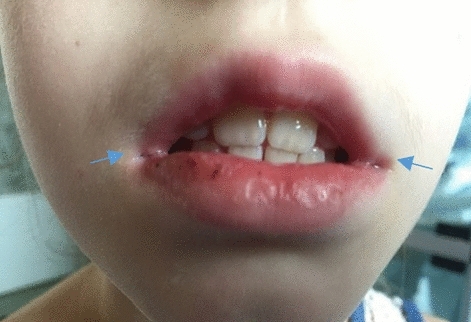
Angular cheilitis in a child diagnosed with ulcerative colitis.

In the study group, 32 (65.4%) children were diagnosed with UC and 17 (34.6%) children with CD. Oral mucosa nonspecific of IBD lesions were found in 18.7% the children with UC and 35.3% the children with CD (Table 4). There was no statistical significance for the occurrence of changes in the oral mucosa between children with UC and CD (Table 4). In the children with CD, the most frequently observed lesion of the oral mucosa was aphthous stomatitis (23.5%) (Fig. 1), and in children with UC, angular cheilitis (12.5%) (Fig. 2). Differences in the type of lesions in the oral mucosa for UC and CD were not statistically significant (Table 5).

**Table 4:** Frequency of occurrence nonspecific of IBD oral changes in the study group.

The occurrence of changes	Ulcerative colitis		Crohn's disease		*p*
	n	%	n	%	
No	26	81.3	11	64.7	n.s
Yes	6	18.7	6	35.3

**Table 5:** Characteristics of changes in the oral mucosa in the study group.

Type of changes	Ulcerative colitis		Crohn's disease		*p*
	n	%	n	%	
No changes	25	78.1	11	64.7	n.s
Herpes	1	3.1	0	0.00	
Aphthae	2	6.3	4	23.5	
Angular cheilitis	4	12.5	2	11.8	

## Discussion

Inflammatory bowel disease is a group of gastrointestinal disease that affects the entire digestive tract from the mouth to the anus. Their etiopathological mechanism is multifactorial, and the symptoms of the disease can develop in both children and adults. More scientific reports describe changes in the course of IBD in adults than in children. The prevalence of the oral lesions in IBD ranges from 0.7% to 37% in adults and from about 7% to 23% in children. In own research, this percentage was 24.49%. Symptoms in the oral cavity can occur either concomitantly with intestinal symptoms or before presenting signs, due to intestinal malabsorption or induced by pharmacological treatments^10^. Oral manifestations of IBD can be specific or nonspecific. In the own research, only nonspecific changes on the oral mucosa were found. Non-specific oral lesions occur more frequently than specific lesions, so differential diagnosis can be difficult.The most common nonspecific manifestation is the oral aphthae. Aphthae, inflammation in the oral mucosa may appear as an isolated oral disease or accompany systemic disease. Aphthae may occur in the course of different systemic disease diseases such as: inflammatory bowel disease, Behçet disease, autoinflammatory syndromes-related oral mucosal changes, anemia, nutritional deficiencies, celiac disease and HIV infection^1^. In our study the prevalence of oral mucosa inflammation in children with IBD was 12.2% both for aphthous stomatitis and angular cheilitis (Table 1). In the control group aphthae and inflammation of the corner of the mouth were less frequent happen but the differences in the occurrence between groups were not statistically significant *p* = 0.07 (Table 3). The study of other authors indicate that in pediatric patients with IBD one of extra-intestinal manifestations (EIMs) is aphthous stomatitis and it prevalence shows a large variability from 3.2 to 41.7%^9^. Younger children at diagnosis have lower EIMs rates compared with older children^11^. Oral mucosal changes may be related to the implemented pharmacotherapy of the underlying disease, coexisting malabsorption syndrome, deficiencies of micro- and macroelements and vitamins (especially vitamin B12) or dry mouth^2,7,12^. Pathogenesis of oral manifestations of IBD remains unclear. Currently research reported on the potential role of microbiota in the pathogenesis of IBD and its oral location, it has been proposed that dysbiosis (term that means imbalance within the bacterial community) of salivary microbiota (with relative abundance of Streptococcus, Prevotella, Haemophilus, and Veillonella) may play a crucial role^13^. Severe IBD has a less diverse microbiota with fewer commensal microbiota communities and more opportunistic pathogenic bacteria originating from the oral cavity or respiratory tract^14,15^. The image of specific and unspecific manifestations of inflammatory lesions within the oral cavity in inflammatory bowel diseases is described as oral mucosa hypertrophy, often with erosions, swelling and ulceration of the lips, inflammation of the corner of the mouth, as well as an overgrowth of the cheek mucosa with its characteristic "paving"^8,16^. Angular cheilitis is characterized by erythema at the corners of the mouth with or without painful fissures and sores. It can be a consequence of anemia or fungal and bacterial infections^13^. In our research prevalence of unspecific inflammatory manifestations in the oral cavity were (35.3%) for children with CD and (18.7%) for children with UC (Table 4). In their studies, Pittock and Harty found similar changes in the oral mucosa more than 40% of children with Crohn's disease^8,12^. Similar results were obtained in study Kłaniecka et all, more than one third of all children with CD had presented inflammatory changes in the oral cavity^17^. Gingival hypertrophy and aphthous stomatitis according to the studies by Dunlap et al., is more common in patients with CD than in UC^18^. This relationship has been confirmed in our research, 23.5% children with CD had the aphthous stomatitis and only 6.3% children with UC (Table 5). The characteristics of the lesions of the oral mucosa in the study group showed that aphthous stomatitis occurred twice as often (23.5% of children with CD) than angular cheilitis (11.8%) (Table 5). While, among children with UC, angular cheilitis was two times more frequent (12.5%) than aphthous stomatitis (6.3%). These differences were not statistically significant.

The problem of the condition of the dentition and the increased risk of tooth decay in children and adolescents with IBD is rarely discussed in the literature. Studies on the relationship between dental caries and IBD are not unequivocal. Some authors point to the greater intensity of caries in patients with IBD, explaining that higher mean DMFT values are a consequence of an increased consumption of cariogenic carbohydrates during the disease as well as a change in the oral bacterial flora (an increase in the number of S. mutans and Lactobacili bacteria). spp.)^19,20^. The studies by Kłaniecka and Kaczmarek, Waśko -Czopik et al. proved no significant difference in the intensity of caries in sick compared to healthy people^7,17^. In our own research, no greater severity of caries was found in children from the study group compared to children from the control group, both expressed by the DMFT index and its P component—active caries (Table 1).

The relationship between inflammatory bowel disease and gingivitis was observed in the studies by Kłaniecka and Kaczmarek, where the study group consisted of children and adolescents aged 3.5–18 years with diagnosed CD (34 individuals) and UC (14 individuals) and also patients undergoing diagnosis (4 individuals). The value of the modified bleeding index (m—SBI) was 2.4 times higher in patients of IBD than in the control group^17^. In our study, gingival bleeding was also almost two times more common in children with IBD (14.3%) than in the healthy group (6.6%). In our own research, in children with IBD, there were no statistically significant changes indicating the occurrence of gum bleeding and tartar compared to the control group (Table 2).

One of limitations of this study is number of the IBD participant's. The reason was that mostly participants were recruited from newly diagnosed patients in one hospital ward and not all parents gave their written consent for their children to participate in the study.

The specific nature of the response make the oral cavity a useful source of biomarkers to diagnose and monitor treatment outcomes in IBD patients^21^. The oral microbiome dysbiosis associated with IBD should be the subject for future research. Physicians examining children and adolescents, especially dentists, should pay attention to the presence of mucosal hyperplasia, erosions or ulcers, inflammatory changes in the lips and angles, which may be an early sign of inflammatory bowel disease, both in the medical history and clinical examinations. Finding in the oral cavity repeating changes should be the reason for referring the patient to a medical specialist. Changes in the oral cavity occurring in the course of Crohn's disease and ulcerative colitis are chronic in nature with periodic exacerbations and are therefore a diagnostic and therapeutic problem requiring both local and systemic treatment. The results of the study show that oral manifestations are more common in children diagnosed with IBD than healthy children. The most commonly observed changes are aphthas and angular cheilitis, which occurred twice as often in children with inflammatory bowel disease compared to healthy children.

## Conclusions

The appearance of repeated, inflammatory changes in the oral mucosa, inflammation of the lips or the corner of the mouth may be one of the symptoms of inflammatory bowel disease in children and adolescents. Interdisciplinary cooperation between dentists, paediatricians and gastroenterologists in both diagnosis and treatment is essential for early diagnosis and improve patients’ quality of life.
